# Embedded Fragments from U.S. Military Personnel—Chemical Analysis and Potential Health Implications

**DOI:** 10.3390/ijerph110201261

**Published:** 2014-01-23

**Authors:** José A. Centeno, Duane A. Rogers, Gijsbert B. van der Voet, Elisa Fornero, Lingsu Zhang, Florabel G. Mullick, Gail D. Chapman, Ayodele O. Olabisi, Dean J. Wagner, Alexander Stojadinovic, Benjamin K. Potter

**Affiliations:** 1 Division of Biophysical Toxicology, Joint Pathology Center, Silver Spring, MD 20910, USA; E-Mails: duane.rogers@afncr.af.mil (D.A.R.); b.v.d.voet@gr.nl (G.B.V.); elisa.fornero@gmail.com (E.F.); lingsu.zhang@usda.gov (L.Z.); florabel.g.mullick.ctr@mail.mil (F.G.M.); 2 Naval Medical Research Unit Dayton, Wright Patterson AFB, OH 45433, USA; E-Mails: gail.chapman@med.navy.mil (G.D.C.); ayodele.olabisi@dtra.mil (A.O.O.); dean.wagner@med.navy.mil (D.J.W.); 3 Combat Wound Initiative Program, Walter Reed Army Medical Center, Washington, DC 20307, USA; E-Mail: alexander.stojadinovic@us.army.mil; 4 Integrated Department of Orthopaedics and Rehabilitation, Walter Reed Army Medical Center, Washington, DC 20307, USA; E-Mail: Benjamin.k.potter.mil@health.mil

**Keywords:** metal-fragments, depleted-uranium (DU), tungsten, heavy metal tungsten-alloys (HMTA), lead (Pb), improvised-explosive device (IED), explosively-formed projectile (EFP), X-ray fluorescence spectrometry (XRF), inductively-coupled-plasma mass-spectrometry(ICP-MS), confocal laser Raman-microspectroscopy (CLRM), scanning-electron-microscopy energy-dispersive X-ray analysis (SEM-EDXA), elemental analysis

## Abstract

*Background*: The majority of modern war wounds are characterized by high-energy blast injuries containing a wide range of retained foreign materials of a metallic or composite nature. Health effects of retained fragments range from local or systemic toxicities to foreign body reactions or malignancies, and dependent on the chemical composition and corrosiveness of the fragments *in vivo*. Information obtained by chemical analysis of excised fragments can be used to guide clinical decisions regarding the need for fragment removal, to develop therapeutic interventions, and to better anticipate future medical problems from retained fragment related injuries. In response to this need, a new U.S Department of Defense (DoD) directive has been issued requiring characterization of all removed fragments to provide a database of fragment types occurring in combat injuries. *Objectives*: The objective of this study is to determine the chemical composition of retained embedded fragments removed from injured military personnel, and to relate results to histological findings in tissue adjacent to fragment material. *Methods*: We describe an approach for the chemical analysis and characterization of retained fragments and adjacent tissues, and include case examples describing fragments containing depleted uranium (DU), tungsten (W), lead (Pb), and non-metal foreign bodies composed of natural and composite materials. Fragments obtained from four patients with penetrating blast wounds to the limbs were studied employing a wide range of chemical and microscopy techniques. Available adjacent tissues from three of the cases were histologically, microscopically, and chemically examined. The physical and compositional properties of the removed foreign material surfaces were examined with energy dispersive x-ray fluorescence spectrometry (EDXRF), scanning electron microscopy (SEM), laser ablation inductively-coupled plasma mass-spectrometry (LA-ICP-MS), and confocal laser Raman microspectroscopy (CLRM). Quantitative chemical analysis of both fragments and available tissues was conducted employing ICP-MS. *Results*: Over 800 fragments have been characterized and included as part of the Joint Pathology Center Embedded Fragment Registry. Most fragments were obtained from penetrating wounds sustained to the extremities, particularly soft tissue injuries. The majority of the fragments were primarily composed of a single metal such as iron, copper, or aluminum with traces of antimony, titanium, uranium, and lead. One case demonstrated tungsten in both the fragment and the connected tissue, together with lead. Capsular tissue and fragments from a case from the 1991 Kuwait conflict showed evidence of uranium that was further characterized by uranium isotopic ratios analysis to contain depleted uranium. *Conclusions*: The present study provides a systematic approach for obtaining a full chemical characterization of retained embedded fragments. Given the vast number of combat casualties with retained fragments, it is expected that fragment analysis will have significant implications for the optimal short and long-term care of wounded service members.

## 1. Introduction

Wounds caused by improvised explosive devices (IEDs) and explosively-formed projectiles (EFPs) are a principal mode of injury on the modern battlefield in the current conflicts in Iraq and Afghanistan. The simultaneous advances in personal body and vehicular armor, rapid aeromedical evacuation of casualties, and deployment of cutting-edge medical technologies and treatments have significantly contributed to an improved survival rate among wounded American service members [1,2]. The cumulative result of these technological advances has been an unprecedented and substantial military and civilian healthcare population comprised of surviving casualties with devastating war wounds, traumatic amputations, and penetrating and closed traumatic brain injuries. These high-energy blast wounds are characterized by massive zones of injury, including bone, muscle and soft tissue injuries, devitalized tissue, and retained foreign materials of unknown composition. Long-term health risks associated with retained embedded fragments composed of metal, organic, and composite materials (e.g., plastic, ceramic, cement, plaster, glass) have yet to be completely elucidated. U.S. military and International Committee of the Red Cross guidelines have recommended observation of retained fragments that are not readily accessible for removal, *i.e.*, those which would expose the patient to undo risk of morbidity associated with surgical attempts to remove the retained fragments [3]. Recent research suggests the need within the military medical community to characterize the chemical and toxicological properties of embedded fragments, and to have this information readily available to health care professionals.

The toxicity of heavy metals is of paramount importance when contemplating the chemical composition of the embedded fragments. Heavy metals such as lead and depleted uranium (DU) have been used in military munitions and protective armor worldwide [4,5]. However, concern regarding the acute and long-term human health and environmental effects of exposure to lead and DU has forced the military in many countries to explore the possibility of using toxicologically-safer metals with comparable material characteristics. Heavy metal tungsten alloy (HMTA)-based materials (containing 90% to 98% tungsten with some mix of nickel (Ni), iron (Fe), copper (Cu), and/or cobalt (Co)) have therefore been recently introduced as potential replacement metals for military applications [6]. Although the toxicological profiles of many of these separate metals are well known, their internalization as embedded fragments retained in soft tissue is an unusual exposure route which may lead to hitherto unseen toxic effects. Concerns regarding potential long-term effects of retained fragments are indicated by recent *in vitro* (using cancer cell lines) [7,8] and *in vivo* animal studies [9] demonstrating that some tungsten-alloys are tumorigenic. In this regard, it is also interesting to note that while Kalinich *et al*. [9] demonstrated that a particular combination of W:Ni:Co (91:6:3) was highly carcinogenic in F344 rat models; recently published studies by Shuster *et al*. [10] used added controls and a different alloy composition to show that neither W alone nor a W:Ni:Fe alloy causes sarcomas in the same animal model. 

Traditional surgical management of embedded fragments has not required surgical exploration and fragment excision and removal, based on the dogma that retained metal pieces remain inert in tissue and do not cause any long-term adverse effects [11]. However, these views are sustained by anecdotal evidence and antiquated practices persisting due to the lack of systematic collection of longitudinal data. The acute and long-term health risks of this specific type of exposure may be underestimated, and are therefore cause for concern. In addition to the elemental composition of a fragment the chemical species of the metal (*i.e.*, oxidative/valence state, isotopic enrichment, covalent and non-covalent coordination chemistry, *etc.*) can also play a significant role in that metal’s bioavailability and toxicity. Although the metal-toxicology focused on DU, lead, and tungsten-alloys may be the primary consideration, the health concern of non-metal materials, *i.e.*, plastics, fibers and particles, should be also considered. These concerns were recently recognized by the U.S. Department of Veterans Affairs (VA) and the U.S. Department of Defense (DoD), and are now being addressed by programs requiring longitudinal surveillance of veterans and laboratory analysis of removed fragments. Information gathered through this work will help in the development of potential health risks assessment, and will guide surgical or medical interventions [12]. 

In direct support of these efforts, the Joint Pathology Center (JPC; formerly the Armed Forces Institute of Pathology) near Washington, DC recently became involved in the laboratory analysis of removed fragments from wounded U.S. service members. This paper describes an approach for the chemical analysis and characterization of retained fragments and adjacent tissues, and includes case examples describing fragments containing DU, tungsten, lead, and non-metal foreign bodies composed of natural and man-made composite materials.

## 2. Methods

### Chemical Analysis of Removed Fragments

**Figure 1:**
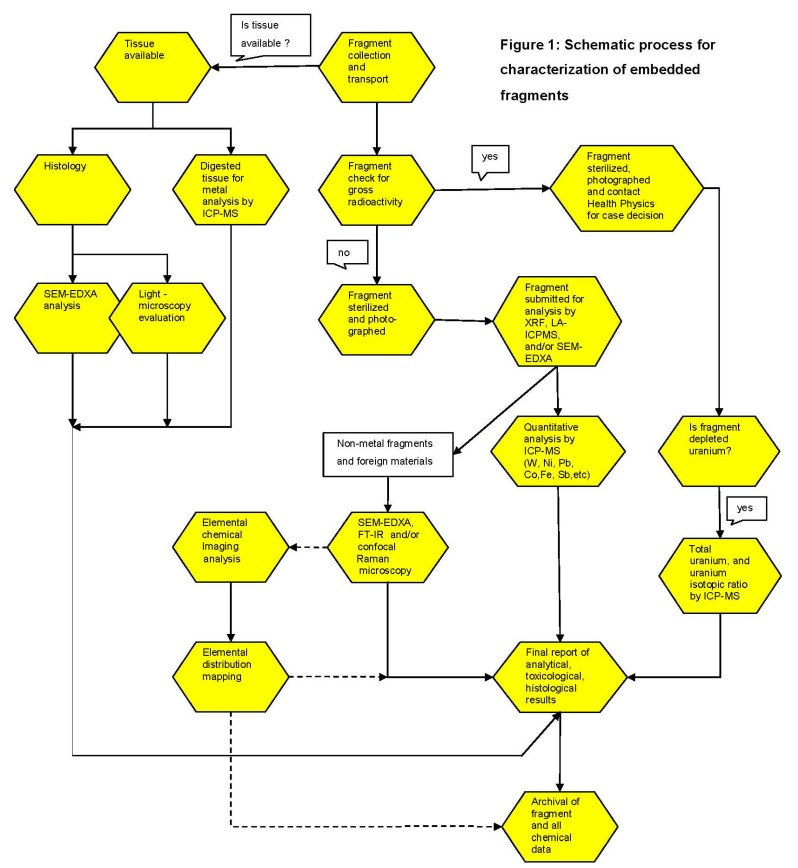
Schematic process for characterization of embedded fragments as performed by the Joint Pathology Center (JPC) Embedded Fragment Registry. The process steps connected by black arrows are always performed; the steps connected by dotted arrows are only taken when considered necessary.

The Joint Pathology Center laboratory protocol for the characterization of removed embedded fragments is shown in Figure 1. The work includes surface characterization, microscopic analysis, and chemical-compositional determination of metal and non-metal fragments. The protocol was reviewed and approved by the Institutional Review Board (IRB) of the JPC.

Scanning-electron microscopy energy-dispersive X-ray Analysis (SEM-EDXA): To determine the microscopic physical appearance and elemental composition of each fragment, a Hitachi S-3500N scanning electron microscope (SEM) (Pleasanton, CA, USA) equipped with a ThermoNoran energy dispersive X-ray analysis (EDXA, Middleton, WI, USA) accessory was used. SEM-EDXA yields information concerning the elemental composition of the fragment but does not allow molecular characterization of non-metallic fragments. 

Confocal Laser Raman-microspectroscopy (CLRM): A LabRam Jobin Yvon (Horiba, Edison, NJ, USA) micro-spectroscopy system equipped with a He/Ne laser operating at 632.82 nm was used to obtain molecular, surface, and structural information of organic and non-metallic composite materials (*i.e.*, plastics, ceramic, cement, plaster, glass, silica, *etc.*), and for the identification of foreign materials in tissue sections. The Raman spectra were recorded at magnification 10× and 100× and processed using LabSpec 4.02 software as previously described [13,14].

Inductively-coupled-plasma mass-spectrometry (ICP-MS) and Energy-dispersive-X-ray Fluorescence Analysis (EDXRF): Quantitative and qualitative chemical analysis was conducted employing several trace elemental analytical techniques. A LSX-500 266 nm laser ablation (LA) system (CETAC Technologies, Omaha, NE, USA) interfaced to a quadrupole inductively-coupled-plasma mass-spectrometer (ICP-MS) was used for elemental characterization of fragments. Laser-ablation inductively-coupled-plasma mass spectrometry (LA-ICP-MS) is a highly sensitive technique that provides semi-quantitative elemental information without the need to digest the specimen. LA-ICP-MS provides a point measurement of elemental composition with a spot size down to 10 µm and only consumes a few picograms of material per measurement. The laser can be rastered [rastor-based images revolve around editing pixels rather than vector-based images that revolve around editing lines and shapes] across the surface of a specimen to provide spatially resolved elemental information or the laser can drill down into a sample to provide an elemental depth-profile when such information is desired. 

Qualitative and semi-quantitative analysis was also performed with energy dispersive X-ray fluorescence spectrometer (EDXRF; ARL QUANTX EDXRF Thermo Scientific, Waltham, MA, USA). EDXRF is a surface characterization, non-destructive technique capable of analyzing light and heavy elements in essentially any type of sample including solids, liquids, powders, tissues, and plastics.

For quantitative elemental composition, fragments, and tissue specimens are digested in nitric acid and analyzed employing an inductively coupled mass spectrometry with quadrupole (Elan 6100 DRC, Perkin Elmer, Waltham, MA, USA) or sector-field (ICP-MS, Element 2, Thermo Scientific) mass spectrometer. Typically 0.2–1 g of fragment material was collected for each sample and digested in concentrated nitric acid (HNO_3_) in a microwave accelerator system (Mars Express, CEM Corporation, Matthews, NC, USA). All analyses were performed in duplicate. Forty elements were measured by ICP-MS using standard operating procedures under strict quality control and quality assurance procedures. Quality control procedures were based on the use of standard reference materials of known composition obtained from institutions such as the National Institutes of Standards and Technology, NIST, Gaithersburg, MD, USA). In addition, quality assurance procedures, including a strict system of daily instrument performance checks, laboratory participation on proficiency testing programs, *etc.*, were used in order to assure and improve the quality of the chemical testing, analysis and information. Detection limits for most of the metals were in the range of 0.1 to 1 ppb (ng·g^−1^), but conventionally results are reported in the ppm (mg·kg^−1^) range.

## 3. Results

### 3.1. Embedded Metal Fragment Registry

Between 9 February 2006 and 30 November 2012, the JPC received 831 fragments from 344 patients. Tissue materials were submitted for 17 of these patients. From 183 patients between one and five fragments per patient were collected. Twenty-seven patients had between five and 10 fragments removed and more than 10 fragments per patient were collected from six patients. For patients presenting with more than five fragments, the most representative fragments were selected based on macroscopic examination, and five to 10 fragments per patient were analyzed. 

The chemical composition of 831 of these fragments is listed in Table 1. Non-metallic materials (*n* =112) included samples of geologic nature and organic-based fragments, such as polymers (*n* = 12), fabric (*n* = 2) and wood (*n* = 2). Most of the fragments consisted of metals (*n* =719), either a single element or a mixture of metals as alloys. Some fragments, such as plastic clad wire (*n* = 2), were counted as both organic and metallic. Predominant among these were fragments composed primarily of single metals such as iron (Fe), copper (Cu), or aluminum (Al), with traces of antimony (Sb), titanium (Ti), chromium (Cr), and lead (Pb). Only one fragment was found to contain tungsten with trace levels of lead and titanium. One case out of the 344 cases was found to be composed of 80% uranium (U). Isotopic ratio analysis identified this fragment as being DU, with trace levels of aluminum, copper, and zinc. Although most fragments were received without associated tissues or any other clinical material, three patients, all males for which tissues were available, were studied by histological and elemental analysis to compare the tissue metal levels with fragment analysis. 

### 3.2. Case Studies

We selected four patients with the corresponding case material from the JPC Embedded Metal Fragment Registry (Table 1). Patients were males with ages ranging from 24–40 years, penetrating wounds, and retained fragments in the soft tissues. Removal of the fragment ranged from a few days (case number 1 and 3), 14 months (case number 4), to 14 years (case number 2) after original injury. 

**Table 1:** Composition of fragments in the Joint Pathology Center Embedded Metal Fragment Registry.

	Number of Fragments
Total number of fragments received:	**831**
Non-Metals:	**112**
Organic materials	26
Plastic and polymers	12
Fabric	2
Wood	2
Geological (rocks, stones)	10
Others	60
Metals:*	**719**
Fe (>50%)	274
(10%–50%)	37
Cu (>50%)	174
(10%–50%)	27
Al (>50%)	76
(10%–50%)	24
Zn (>50%)	26
(10%–50%)	36
Pb (>50%)	15
(10%–50%)	1
Ni (>50%)	1
(10%–50%)	14
Cr (>50%)	1
(10%–50%)	12
DU ** (>80% U)	1

* Fragments were categorized as containing >50% and 10-50% of the listed element on a weight basis. ** DU = Depleted uranium.

#### 3.2.1. Case Number 1

**Figure 2:**
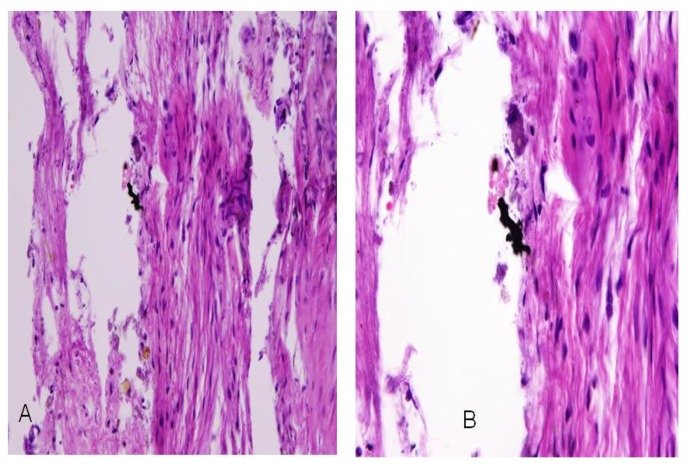
Case 1: Hematoxylin & eosin (H&E) stained section of fibroadipose tissue showing black granular material in aggregates. Original magnifications at 10× and 600×.

**Figure 3:**
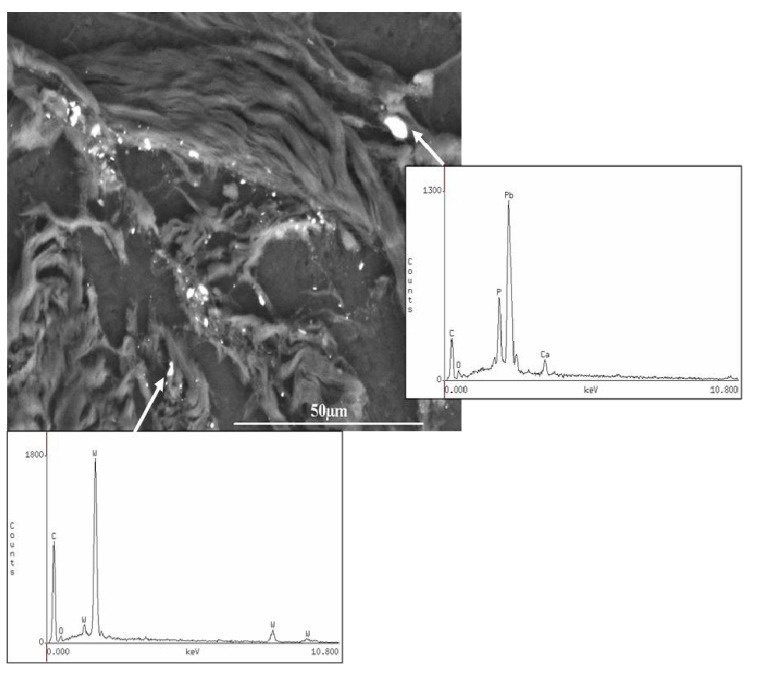
Case 1: Scanning electron microscopy (SEM) image and energy-dispersive X-ray analysis (EDXA) of the granular material embedded in the fibroadipose tissue demonstrating the presence of tungsten and lead. SEM original magnification 900×.

A retained fragment in the left index finger of a 32-year-old male service member as a result of an IED explosion was submitted for analysis. The material submitted contained fibroadipose tissue and three separate fragments. The adherent tissue material was processed for light microscopic histology examination (hematoxylin & eosin—H&E) and for SEM-EDXA analysis on carbon disk sections. The H&E section demonstrated black granular material that was clustered in aggregates (Figure 2). By SEM-EDXA, this black granular material was attributed to tungsten and lead (Figure 3). 

The distribution of tungsten was not uniform throughout the tissue. Rather, only single particles, measuring between 1 to 2.5 µm, showed detectable levels of tungsten under the SEM-EDXA. Similarly, lead was detected in single particles within the tissue, but was not associated with the tungsten. The presence of iron (data not shown) within the tissue section was associated with phosphorus and calcium, suggesting secondary calcium phosphate deposition, presumably at a site of prior hemorrhage adjacent to the foreign metallic material (unpublished observations).

Selected sections of the fibroadipose tissue were chemically digested and further analyzed by ICP-MS. The results demonstrated high levels of copper (0.12% *w*/*w*), iron (2.6% *w*/*w*), lead (0.8% *w*/*w*) and tungsten (0.08% *w*/*w*) within the adipose tissue and correlated very well with the SEM-EDXA findings on the tissue section. By SEM-EDXA, each of the three additional metal fragments demonstrated tungsten and lead, as well as copper and iron, which was further confirmed by ICP-MS. 

#### 3.2.2. Case Number 2

A 40-year-old (male) 1991 Gulf War veteran was injured inside a vehicle reinforced with depleted uranium. He sustained diffuse fragment injuries to his bilateral lower extremities. After 12 years of relatively constant urine uranium levels (as detected through the evaluation of clinical data from the VA DU Clinical Surveillance Program), the patient began demonstrating rising levels of U in his urine without evidence of additional trauma to his lower extremities, or clinical symptoms associated with these fragments. As a preventive measure, the fragments were removed in an attempt to reduce the urine U levels. The excised fragments and the associated fibroconnective tissue were all submitted for histologic, microscopic, and chemical analysis. 

**Figure 4:**
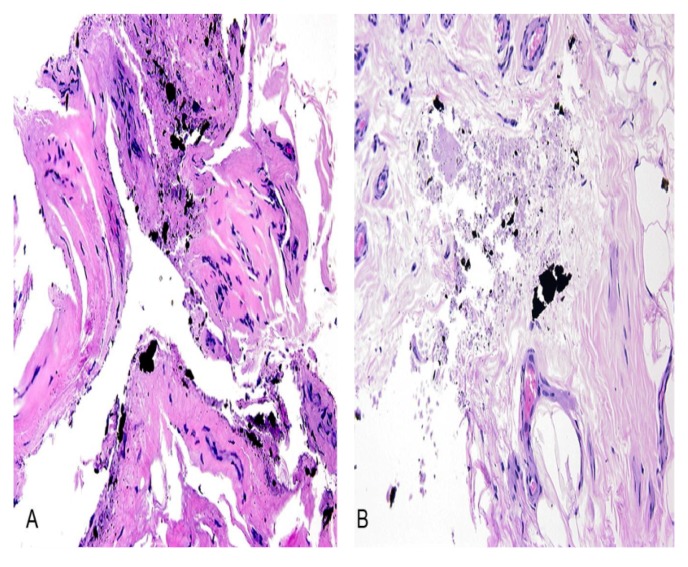
Case 2: Hematoxylin & eosin (H&E) stained section containing (**A**) pausicellular fibrous tissue and scattered black fragments and (**B**) necrotic debris admixed with black material, but no significant inflammation. Original magnification 200×.

The H&E stained sections were examined by light microscopy, and demonstrated dense fibrous tissue (capsule) surrounding the fragment composed of paucicellular fibrous tissue and scattered black metal fragments (Figure 4A). Furthermore, the histologic analysis demonstrated some focus of necrotic debris admixed with the black material, but no significant inflammation was detected (Figure 4B). 

**Figure 5:**
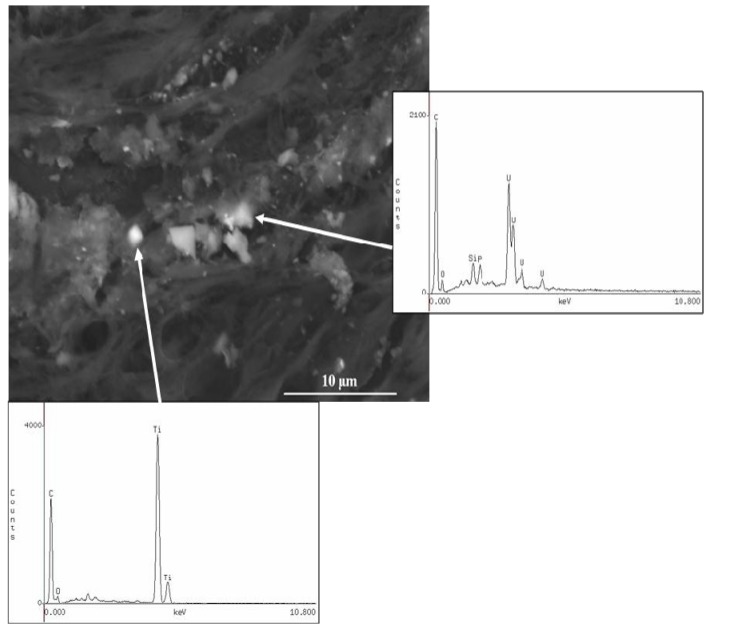
Case 2: Scanning electron microscopy (SEM) image of the DU fragment and energy-dispersive X-ray analysis (EDXA) of a region of tissue with granular black fragments demonstrating the presence of uranium and titanium. Depleted Uranium particles were measured in in the range of 0.8–1.69 µm. SEM original magnification 3,000×.

Special stained sections for etiologic infectious microorganism were negative. By SEM-EDXA analysis of the tissue sections, the granular black fragments demonstrated both uranium and titanium (Figure 5). Quantitative analysis of the fragments for total uranium was conducted using quadrupole-based ICP-MS, while depleted uranium was analyzed by measuring the ^235^U/^238^U employing a high-resolution (sector-field) ICP-MS as previously described [15,16]. Measurement of uranium isotopic ratios was used to characterize the uranium as either natural uranium (*i.e.*, ^235^U/^238^U = 0.00725), depleted uranium (*i.e.*, ^235^U/^238^U = 0.0022), or enriched uranium (*i.e.*, ^235^U/^238^U = 0.009). Results of the ICP-MS analyses are provided in Table 2. Although results are reported in the ppm (mg/kg^−^^1^) range for many of the elements listed on Table 2, detection limits (DL) were in established in the range of 0.1 (for Cu) to 1 ppb (ng·g^−1^) (for Mg) as 3× the standard deviation of the blank signal. For W, our detection limit was determined as 1.5 ng/g, while a DL of 10 ng/g was established for Al. Isotope-ratio analysis indicates that all three fragments contained DU as demonstrated by an isotopic ratio of 0.002.

DU is the residual metal remaining after the uranium enrichment process which depletes or lowers the relative concentration of the ^234^U and ^235^U isotopes, leaving 99.8% in the form of ^238^U with lower specific activity. The ICP­MS isotopic ratio of ^235^U/^238^U demonstrated unequivocally that the main chemical constituent of the fragments in this case is DU, while trace amounts of other metals including titanium, chromium, and aluminum were also detected. Each fragment, as well as the tissue sections, was analyzed employing SEM-EDXA, and a significant correlation was found between the uranium and titanium present in the tissues and the fragments. Figure 5 illustrates the SEM photomicrograph of a region of tissue removed close to fragment 1, demonstrating uranium and titanium containing particles as determined by SEM-EDXA. The particle sizes were in the range of 0.3–2.5 µm. 

**Table 2:** Elemental composition of uranium- and depleted uranium-containing fragments (case number 2)

Element	Fragment #1 (µg/g, ppm)	Fragment #2 (µg/g, ppm)	Fragment #3
U	204,179	15,659	924,872
^235^U/^238^U ***	0.0022 *	0.0023 *	0.0022 *
W	194	81	<DL **
Ti	1,993	199	1,340
Cu	378	112	12
Al	738	15,030	<DL
Mg	305	517	14

* Uncertainty = ± 0.0001; ** DL = Detection limit (3SD of blank signal); *** Depleted uranium interpretation is based on the result of the uranium isotope ratio for ^235^U/^238^U as: a. Natural uranium = 0.0060–0.0090, average = 0.0073; b. Depleted uranium = 0.0020–0.0026, average = 0.0022.

#### 3.2.3. Case Number 3

**Figure 6:**
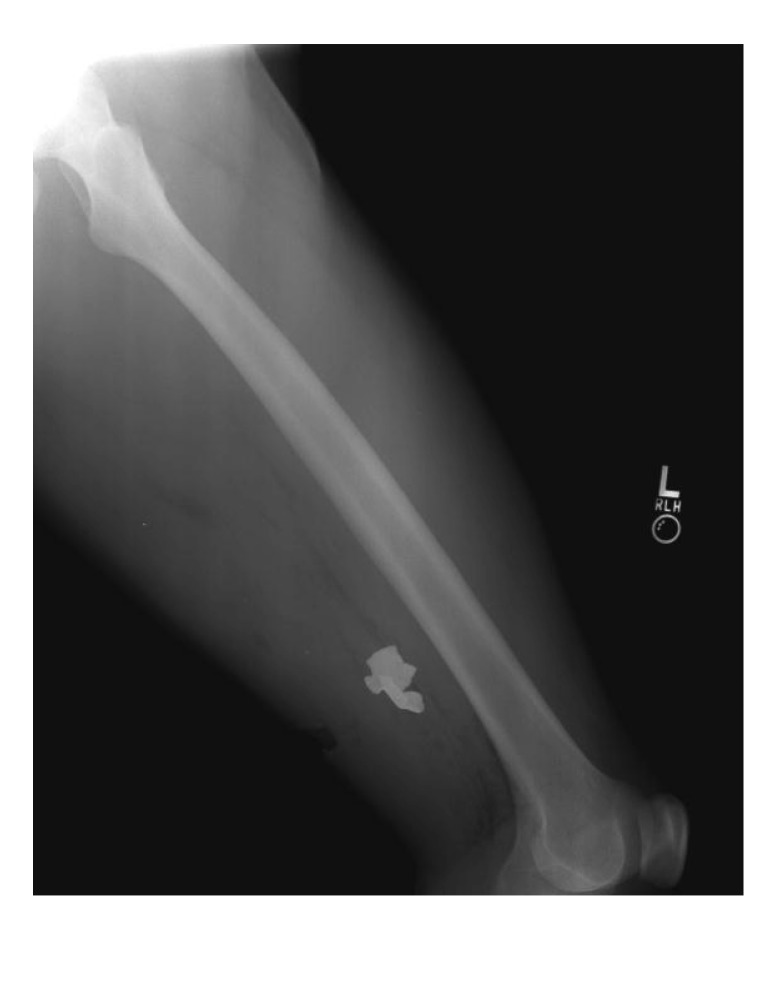
Case 3: Lateral radiographs of the left thigh and femur of a 24 year-old male service member demonstrating opacities representing retained metal and composite material fragments in the soft tissues of the posterolateral thigh due to an improvised explosive device blast.

**Figure 7:**
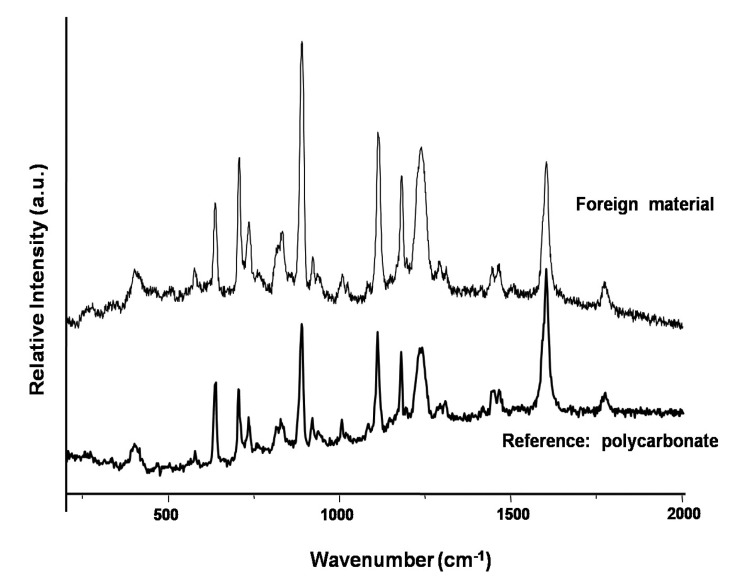
Case 3: Confocal laser Raman microprobe spectrum of a non-metallic fragment identified as bisphenol-A-polycarbonate.

Case 3, is a 24-year-old male with retained fragments from a wound to the lower extremity (Figure 6). Two fragments were submitted. No tissue was available for analysis. One fragment was found to be metallic, predominantly composed of iron as demonstrated by SEM-EDXA (data not shown). Calcium, phosphorus, and oxygen were also detected suggesting secondary calcium phosphate deposition at the site of encapsulation. A second fragment was determined to be a polymer identified as bisphenol-A-polycarbonate (CAS Number: 25037-45-0) by confocal laser Raman microspectroscopy (CLRM) (Figure 7).

#### 3.2.4. Case Number 4

**Figure 8:**
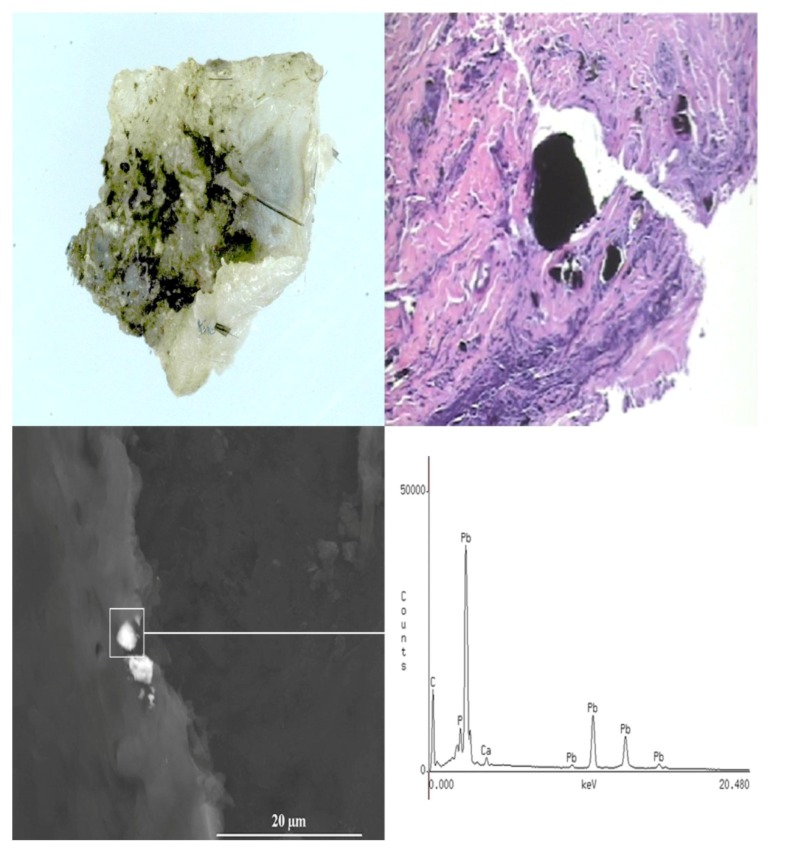
Case 4: Gross photograph (**A**) of a retained fragment (black area) surrounded by fibroadipose tissue (white area); (**B**) Hematoxylin & eosin (H&E) stained section demonstrating granular black fragments. Scanning electron microscopy image and EDXA spectrum (**C**) of a tissue section demonstrating the presence of a metallic lead fragment within the fibroadipose tissue.

A 25-year-old male with a fragment injury to the back of the head and shoulder from a gunshot wound, suffered in 2007 while serving in Iraq, was treated, but small fragments were retained in the soft tissue scalp of left parietal/temporal region, left inferior occiput, and left posterior shoulder overlying scapula. At follow-up a year later, the patient requested excision of the fragments. A wound fragment measuring 0.7 cm × 0.5 × 0.1 cm was excised from the posterior neck and submitted for analysis. A gross photograph of the removed fragment (irregularly shaped and black in color) surrounded by soft tissue is shown in Figure 8A. The H&E stained section demonstrate black fragments scattered throughout the tissue (Figure 8B) including birefringent (birefringence is responsible for double refraction whereby a ray of light is split by polarization into 2 rays taking slightly different paths when incident upon a birefringent material) foreign bodies. By SEM-EDXA, the black scattered fragments were identified as being composed of lead, in both the excised fragment and the surrounding tissue (Figure 8C). 

Quantitative analysis of the tissue by ICP-MS demonstrated a level of 11% (*w*/*w*) Pb in the surrounding tissue. These high levels of lead within the digested tissue suggest that micro-particles of lead remained in the tissue specimen even after the fragment was carefully removed from the adherent fibroadipose tissue. The lack of any other metals on both the SEM-EDXA and ICP-MS analyses indicate that this embedded fragment is made of metallic lead. 

**Figure 9:**
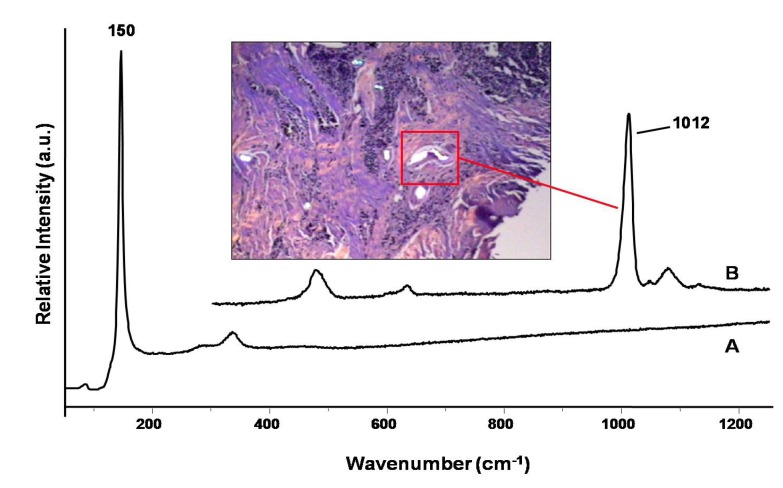
Case 4: Confocal laser Raman microspectroscopy spectra demonstrating the presence of PbO (trace A) and CaSO_4_ (trace B) within a tissue section.

The tissue section was further studied employing confocal laser Raman microspectroscopy (CLRM). This technique is a non-destructive approach which can be used for the characterization of compounds, composite and minerals that may be present as foreign materials in tissue sections. A 5-µm thick section of paraffin-embedded tissue was mounted on an aluminum-coated microslide, deparaffinized by washing with xylene, and examined by CLRM. As shown on Figure 9, Trace A is a Raman spectrum with peaks at 150 and 350 cm^−1^, which are attributed to the formation of lead(II) oxide (PbO) at the site of the black fragment [17]. To our knowledge, this is the first time that this form of lead has been demonstrated by Raman microspectroscopy to be formed in tissues as a result of exposure to lead. Adjacent to the metal fragment, a deposition of brown to dark refractive foreign bodies were observed (see the insert on Figure 9). These inclusions were identified by Raman microscopy (Trace B) to be calcium sulfate (CaSO_4_) deposition (Raman peak of 1,012 cm^−1^), which commonly occurs near retained fragments in response to the presence of the foreign material. 

## 4. Discussion

The recent directive established by U.S. Department of Defense requiring a comprehensive analysis and characterization of metal fragments removed from DoD personnel is a first important step in establishing appropriate health care for personnel with retained, potentially hazardous, embedded fragments [12]. The presented manuscript describes procedures established at the JPC to develop a registry for the analysis, characterization, and archival of embedded fragments and adjacent tissue, using four representative cases.

Reasons for surgical removal of embedded fragments include their clinical and radiological accessibility, proximity to neurovascular structures, fragment size and perceived secondary infection risk due to the colonized retained foreign body, location in weight bearing surfaces (e.g., palms, feet) potentially causing pain or damage, and intraarticular location causing risk of arthritis [18,19,20,21,22,23,24]. Traditional views on surgical management of embedded fragments, however, do not generally call for surgical excision and removal, and are often based on the consideration that metal pieces do not cause any long-term damage [11,12]. This view may be built on practical experience and relatively short clinical follow-up, but is certainly not supported as yet by long-term clinical and laboratory observations.

From this work, metals are the most predominant constituents of fragments removed from personnel injured by IEDs or EFPs. Other foreign materials identified as embedded fragments may consist of chemically pure polymers, glass, wood, rocks, and other non-reactive inert materials. Iron, copper, and aluminum are the main components of the majority of the collected fragments. Moreover, most of the fragments contain far more than one metal. The toxic metals lead, tungsten, and DU were identified in only a few fragments. The short-term objective of the chemical characterization of the fragments is to provide immediate clinical guidance. The presence of tungsten or uranium in a fragment may contribute to a surgeons’ decision to clinically manage cases with retained fragments, by developing biomonitoring testing, or by further removal or observing remaining fragments.

The long-term objective of the chemical characterization is the informed biomonitoring and follow-up of veterans living for years to come with embedded fragments in their bodies. The majority of the fragments are expected to remain encapsulated and inert at a certain site in the body for a long period of time. A number of fragments, however, will undergo a process of gradual degradation and function as a slow-release depot giving rise to a chronic systemic exposure. Moreover, potential fragment migration, even after many years, cannot be excluded, as the migration of retained medical implants to remote sites has been reported with relative frequency [25,26,27]. Regular X-ray follow-up and regular monitoring of metals in blood or urine may keep track of these processes and may help to determine health risk and intervention if necessary [11]. 

Although the development of malignancies and autoimmune diseases associated with foreign bodies (both metallic and non-metallic) is viewed as a rare event in humans, there are a number of case reports describing such conditions in humans long (years) after an incident [28]. Cases such as malignant fibrous histocytoma presenting 44 years after penetrating arm injury [29], sclerosing cholangitis secondary to retained fragment [30], non-malignant pseudotumor arising 61 years after steel grenade fragment implantation [31], angiosarcoma arising 63 years after implantation of grenade fragments [32], and squamous cell carcinoma arising in skin overlying a retained bullet (containing lead with a steel core), with a chronic draining sinus [33], are among some of the reported cases involving retained fragments. It is suspected that such cases are underreported. Moreover, the use of DU and tungsten alloys in the latest decade causes concern. DU and some tungsten alloys such as W:Ni:Co have been shown to be tumorigenic in cultured cells and in experimental animals [7,8,9]. The incidence of malignancies associated with embedded fragments may increase if timely intervention does not occur.

## 5. Conclusions

Blast injuries can lead to multiple retained fragments of unknown composition. Evidence suggests that embedded metal fragments in the body can pose significant local and systemic health effects. Therefore, the study of the chemical composition of retained fragments, and the documentation of the health effects associated with these materials observed via long-term clinical and laboratory follow-up is needed. Knowledge learned will support clinical monitoring programs and timely intervention for combat-wounded service members, as well as civilian nationals, with both removed and retained embedded fragments.

## Disclaimer

This manuscript has been reviewed in accordance with the policies and guidelines of the Joint Pathology Center and the Department of Defense, and approved for publication. Approval should not be construed to reflect the views and policies of the Department of the Army, the Department of the Navy, the Department of Defense, or the United States Government, nor does mention of trade names or commercial products constitute endorsement or recommendation for use. Copyright transfer for work conducted by US Federal Government scientists using US Federal Government facilities, may not be available with this manuscript.
